# A year in words: The dynamics and consequences of language experiences in an intervention classroom

**DOI:** 10.1371/journal.pone.0199893

**Published:** 2018-07-06

**Authors:** Lynn K. Perry, Emily B. Prince, Adriana M. Valtierra, Camila Rivero-Fernandez, Mary Anne Ullery, Lynne F. Katz, Brett Laursen, Daniel S. Messinger

**Affiliations:** 1 Department of Psychology, University of Miami, Coral Gables, FL, United States of America; 2 Linda Ray Intervention Center, University of Miami, Miami, FL, United States of America; 3 Department of Psychology, Florida Atlantic University, Fort Lauderdale, FL, United States of America; 4 Department of Pediatrics, University of Miami, Coral Gables, FL, United States of America; 5 Department of Electrical & Computer Engineering, University of Miami, Coral Gables, FL, United States of America; 6 Department of Music Engineering, University of Miami, Coral Gables, FL, United States of America; Leiden University, NETHERLANDS

## Abstract

Children from low SES backgrounds hear, on average, fewer words at home than those from high SES backgrounds. This word gap is associated with widening achievement differences in children’s language abilities and school readiness. However relatively little is known about adult and child speech in childcare settings, in which approximately 30% of American children are enrolled. We examined the influence of teacher and peer language input on children’s in-class language use and language development in an intervention classroom for low-SES, high-risk 2- to 3-year-olds. Over the course of a year, day-long recordings of the classroom were collected weekly with LENA recorders. Using LENA software algorithms, we found that language input from peers was positively related to children’s in-class language use, both in-the-moment and over the course of each day, as were the number of conversational turns in which children and teachers engaged Both peer input and conversational turns with teachers were also positively related to children’s language development rates, as indexed by increases in vocabulary size. Together these results indicate the importance of child-specific rates of classroom language input in the language development of high-risk, preschoolers.

## Introduction

Language input from caregivers has cascading consequences for child development. By 3 years of age, children from lower SES backgrounds are estimated to hear 30 million words fewer than their peers from higher SES backgrounds [1]. These differences in input predict differences in vocabulary knowledge, school readiness, and later academic achievement [2–5]. Relatively few studies, however, have examined language input in out-of-home childcare settings, especially with respect to consequences for children’s own language use and development. To address this gap, we examined the role of language in early childcare settings from a high-risk, low-SES sample of preschool-aged children. Recent technologies such as those incorporated in the Language ENvironment Analysis (LENA) system, provide reliable estimates of children’s language input and use [6–11], allowing for efficient collection of day-long recordings and increasing the feasibility of simultaneously studying multiple children’s experiences in childcare settings. Here, we use LENA to document children’s language input from teachers and peers during a year in an early intervention childcare program. We investigate the relationships between language input and use, both in the moment and over developmental time.

### Which language experiences matter most for development?

We looked to the research on language input from parents to guide our predictions about which language experiences might matter in the classroom. Research on parents’ language demonstrates the consequences of activity context [8,12,13], language quantity [2–5], and language quality [14–16] on children’s language use in-the-moment and on language development over time. High quality input can be quantified by measuring the extent to which caregivers engage children in conversational turn-taking [17]. When parents expand upon what a child says or ask a follow-up question (“Yes, that is a cup. What color is it?”), they encourage their child to participate in a conversation, increasing the child’s opportunities to learn and say new words. Children whose parents use more high-quality language typically have larger vocabularies than children whose parents use less high-quality language (see e.g., [3,14,15]).

Initial research suggests that caregivers’ language varies in both quantity and quality based on activity context. Hoff found that parents have higher rates of talking during dressing and reading than other activities, but higher levels of lexical complexity during mealtime and play [12]. However, the quantity of children’s talking did not differ across settings at home, suggesting that increases in parent talking may not lead to immediate increases in child talking [12]. More recently, Soderstrom and Wittebolle used LENA recorders to compare how language input varied across activities for children at home and in the classroom [8]. In both home and classroom settings, highly structured activities, such as story time or organized play, were associated with increased in-the-moment adult talking, but not increased in-the-moment child talking. Although highly structured activities may not lend themselves to increases in child talking, Roy et al. found that consistency in activity context in a single child’s home was associated with the timing of early word learning [13].

Despite what is known about the effect of language input in the home, the impact of language in classroom settings is less clear. Approximately 30% of American children under 5 attend a childcare center for an average of 36 hours per week [18]. Language input in childcare settings may supplement deficits in language input at home. For example, parents from high SES backgrounds tend to engage in more conversational turns with children than parents from low SES backgrounds, using more expansions and asking more questions [1,3,12,14–16,19]. This variability in home language input may make language input received elsewhere, such as early childcare contexts, more critical. However, the input *individual* children receive in classrooms, both from their teachers and their peers, has not been examined as a potential source of variation in child language development. We investigated how child-specific input from teachers and peers influence children’s classroom language production and language development over time.

### Language experiences in external childcare settings

Although families’ access to high quality care varies, research suggests that high quality preschools with supportive language environments lead to measurable gains in children’s social and communication skills [20–22]. Preschools providing dense exposure to complex vocabulary and syntax—from both teachers and peers—have been shown to positively impact children’s language development over the course of the year [23–25] and later reading abilities even several years after children have entered elementary school (e.g., [26]). Early classroom factors, then, can have long-reaching impacts, which may be particularly valuable for children from lower-SES backgrounds, who tend to be exposed to less language in the home. However, previous research has primarily used entire classrooms as the unit of analysis, asking, for example, how the quality of teacher language is associated with class language performance over multiple classes. While informative, these studies cannot provide an understanding of the day-to-day dynamics of language use within a single classroom—an issue critical to understanding the role of classroom language experiences in children’s development.

### Rationale for current study

The central goal of the current study is to understand the relation between individual children’s classroom language input and language use over moments and over days, and the relation of this input to vocabulary development over the course of a year. We used LENA recorders to estimate children’s language use and input from teachers and peers. Although language is more than vocalizations, LENA estimates of child vocalizations are highly correlated with standardized assessments of expressive and receptive language ability [6]. We use LENA estimates of the quantity of language used by each target child (“child vocalizations”), the quantity of input from peers (“peer vocalizations”) and the quantity (“adult vocalizations”) and quality (“turn-taking”) of input from teachers as measures of language input and use over multiple timescales (i.e., 5 minute, 1 day, and changes over 1 year). We then examine whether language input and use relate to language development, as measured by changes in children’s vocabulary size. Although previous studies have used LENA to demonstrate that the quantity of language children hear at home or in school relates to their later language development, these studies have been relatively short term, encompassing 1 or 2 days [8,11,22,27]. We employ a big data approach of lengthy, repeated measurement of individual children’s language milieus. The goal is to shed light on both in-the-moment vocal interactions and long-term developmental change, and to suggest associations between these timescales. We examine language input from teachers and peer across activities for a year in an early intervention classroom for high-risk children from low-SES backgrounds. The presence of multiple children within the same classroom (same activities, same teachers, same peers) allows us to examine how individual differences in daily vocal interactions are associated with development. The study’s hypothesis is that the input children receive from their peers and teachers will be positively associated with their own language use and development at multiple timescales.

## Methods

This study involved human subjects and was approved by the University of Miami IRB. The project approval number is 20150469. Parents or legal guardians provided consent to participate in the study. We conducted our study in a center-based early intervention classroom for children, ages birth to 3 years, who come from low-SES, at-risk families and met criteria for services under Part C of the Individuals with Disabilities Education Act [28]. Children were referred to the intervention program due to early developmental risk factors. At the time of acceptance to the intervention program, the children in the study all scored at least 1.5 standard deviations below the mean in 2 or more developmental domains, or 2 standard deviations below the mean in 1 or more developmental domains, as measured by the Battelle Developmental Inventory- Second Edition (BDI-2) [29]. At the beginning of the study, all children were below the 30^th^ percentile in expressive vocabulary size [30]. All children qualified for the Department of Health’s childcare food program for free/reduced lunch.

### Participants

Fifteen monolingual English-speaking 2-year-olds were enrolled in the study classroom for at least a portion of the year. Parents or legal guardians provided consent to participate in the study. Children needed to be present on at least 2 recording days to be included in the final sample. See Table 1 for child characteristics. This criterion removed 2 of the fifteen children from analyses: 1 child withdrew from school after 2 months and only contributed 1 recording; another joined the classroom 1 month before the study ended and also only contributed 1 recording. Of the remaining 13 children, 8 were enrolled in the classroom for the entire year; 3 students joined the classroom approximately 6 months into the year; 2 withdrew from school, but not the study, 9 months into the year. Of these final 13, 4 were male. The race/ethnicity composition of the classroom was 70% African American.

**Table 1:** Characteristics of each child in the classroom.

Child	Sex	Overall annual classroom attendance rate	Number of recordings	Age at enrollment (months)	Time 1 vocabulary	Time 2 vocabulary	Time 3 vocabulary
1	female	.61	9	26	11	315	no longer enrolled
2	male	.38	8	28	50	261	no longer enrolled
3*	female	.09	1	23	48	no longer enrolled	no longer enrolled
4	female	.93	18	24	1	87	108
5	female	.64	17	21	83	399	429
6	male	.55	13	26	24	303	435
7	female	.56	17	30	78	318	457
8	female	.50	10	23	24	233	415
9	female	.83	22	28	180	498	475
10	female	.47	10	22	29	91	427
11	male	.13	7	24	15	74	119
12	female	.15	5	30**	not yet enrolled	382	431
13	male	.45	16	28**	not yet enrolled	5	11
14	female	.28	13	36**	not yet enrolled	412	430
15*	female	.05	1	32***	not yet enrolled	not yet enrolled	286

**Notes.** Time 1 expressive vocabulary size was measured 1 month prior to study initiation; Time 2 expressive vocabulary size was measured 6 months into the study; and Time 3 expressive vocabulary size was measured 1 month after study completion.

*child contributed too few recordings and was not included as a participant.

**child was enrolled 6–8 months after study initiation.

***child was enrolled 11 months after study initiation.

### Data collection

Audio from all children was recorded using Language ENvironment Analysis (LENA) Digital Language Processers (DLPs). LENA recordings were collected weekly for approximately 1 year (42 of the 52 weeks to allow for holidays and school breaks). General school attendance varied widely across individual children (*M* = 119 days; *SD* = 56 days; see Table 1). On recording days, there were an average 6 children and 3 adults (a primary teacher, aides, and observer) in attendance. At any time during a recording day there were between 1 (e.g., during personal care activities) and 27 children present (e.g., when they joined another class at recess).

On each recording day, between 2 and 7 children were selected to wear a LENA recorder for the entirety of the school day (increasing numbers of DLPs became available over the school year). Selection was pseudo-random to balance the number of recordings of each child. However, because individual attendance varied, some children contributed more recordings than others (*M =* 12.7 recordings; *SD* = 5; see Table 1). Over the 42 recording days, this yielded 165 individual recordings and over 680 hours of recordings. On recording days, all children in the classroom wore a t-shirt with a specially designed pocket on the front for the LENA recorder, so that both teachers and children were unaware which shirts contained recorders. While it is possible that may have subsequently ascertained which children were wearing recorders, the procedure minimized this possibility. A school psychologist activated the recorders and placed them in the vests when the children arrived at school in the morning. The psychologist removed the recorders when each child’s parent or caregiver arrived for pickup in the afternoon.

### Measures

Our primary questions concern the role of different sources of language input on children’s language use and language development. We quantify each child’s language input from other children (peer vocalizations) and teachers (adult vocalizations, and turn-taking), as well as their own language use (child vocalizations), using the LENA PRO V3.4.0 software system. We quantify language development via changes in expressive vocabulary based on teacher report on the MCDI.

#### LENA audio analysis

Following data collection, audio files were analyzed using LENA signal processing software which distinguishes child and adult speech-related vocalizations from other vocalizations (crying, burping) and from ambient noise in the environment [31]. Coded vocalizations include any phonemic production, from babbling to complete words. The primary measures of interest were LENA estimates of 1) target child vocalization count (the number of vocalizations made by the child wearing the LENA recorder), which we refer to as “child vocalizations”; 2) adult word count, or the number of vocalizations made by teachers to the target child, which we refer to as “adult vocalizations*”*; 3) non-target child vocalization count, or the number of vocalizations to the target child by other children, which we refer to as “peer vocalizations*”*; and 4) the number of conversational turns between the target child and adults, or the number of changes in speaker when the target child and an adult are talking in close temporal proximity with no longer than 5 seconds of silence between utterances, which we refer to as “turn-taking.” Note that this measure of turn-taking only included conversations between adults and children. We also measured LENA estimates of “overlap,” or periods of overlapping vocalizations that could not be reliably assigned to the target child, peers, or adults, which we used as a covariate. Additional details of the LENA system algorithm can be found in technical reports at http://www.lenafoundation.org/research/.

LENA software quantified each of the speech variables in 5-minute bins. We make these data available at https://osf.io/d7akm. The distributions of each of these variables were log-normal, and we transformed data in each 5-minute bin by calculating log_10_ (x + 1). We calculated log_10_ (x + 1) rather than log_10_ (x) to account for zeros in the data (e.g. 5 minute bins with no vocalizations). To obtain measures of daily behavior, we summed adult vocalizations, child vocalizations, peer vocalizations, and turn-taking for each recording session in each of the 5-minute bins for a given day and divided by the length of the recording in hours.

#### Expressive vocabulary

We measured expressive vocabulary via teacher report on the MacArthur-Bates Communicative Development Inventory Words and Sentences form (MCDI) [32]. We used vocabulary size as an index of language development because it is highly associated with other aspects of language ability, such as syntactic complexity [33] and processing speed [34]. Many previous studies examining caregiver input quality and quantity have used the MCDI or other measures of vocabulary size as indications of children’s language growth (see e.g., [1–3,11,15,19]).

Teachers completed MCDI forms for all children enrolled in the class at 3 time points: 1) 1 month prior to the first recording date; 2) approximately 6 months into the study; and 3) 1 month after the final recording date. Although the MCDI was developed as a parent-report measure, many of the children in our study had unstable home lives. Collecting vocabulary data via teacher-report was thought to be more feasible and potentially more accurate than using parent-report. A potential concern is that teacher-report of vocabulary is based on the extent to which a child can be observed talking in class. However, in a subset of the children (n = 9) who completed a Preschool Language Scales Fifth Edition [35] assessment of general receptive and expressive language abilities, total language age equivalent scores were highly correlated with their MCDI vocabulary score (*r* = .72, *p* = .029), suggesting the validity of using teacher-report on the MCDI as an index of language ability.

#### Activity context

On each recording day, a research associate observed the class and coded the beginning and end time of each activity context [similar to the activity codes used in 8]. In our analyses of activities, we removed language that occurred during nap times and transitions, because this code did not represent a specific type of activity and only accounted for a short amount of time per day. Because previous work has shown that highly structured activities are associated with high amounts of parent language, but not child language [8,12], we classified the remaining activities as either “structured” (story time, circle time, and organized play) or “unstructured” (meal time, general play, personal care, and outside play) based on the organization of interactions. Overall, 72% of an average day was spent in unstructured activities and 28% in structured activities.

### Analytic approach

Analyses of vocabulary growth, in which a single index of growth was predicted per child, were conducted using linear regression. All analyses predicting repeated LENA measure of vocalization per child were conducted using linear mixed effects regression models through the lmer function in the lme4 package of R [36]. Mixed effects models generally and lmer specifically are not affected by differences in the number of data points (observations) contributed by each subject [37], which is ideal for the current situation where children’s attendance varies (see Table 1). For the mixed effect models, we report fixed effects coefficients and standard errors, random effects variance, and standard deviations from the full model, and the results of chi-square tests of model fit comparing nested models with and without each of the predictors of interest. To determine appropriate random effect structure, we began with completely specified random effects structures including random slopes for all variables in a given model. Using model comparison, we systematically removed uninformative random effects [38]. All final models included random intercepts for the subject intercept. In these mixed effects models, observations (level 1) were nested within children (level 2). A standard equation may be written as:

$$Y_{ti}=\gamma_{00}+\gamma_{10}X1_{ti}+\gamma_{20}X2_{ti}+\gamma_{30}X3_{ti}+\varepsilon_{ti}$$

where Y_*ti*_ is the number of child vocalizations for the *t*th observation of the *i*th child, *γ*_00_ is the intercept and random variance in this intercept allows for child-specific variability in vocalization levels, *γ*_10_ is the adult vocalization coefficient, *γ*_20_ is the peer vocalization coefficient, and *γ*_30_ is the turn-count coefficient. Coefficients for sex and age are not shown. *ε_ti_* is the level 1 residual terms with mean of 0 and variance of σ^2^. All model effects are reported in Tables 2–6. Model details are listed in the Appendix and referenced by number in the main manuscript (e.g., “See part M1a of the Appendix”).

**Table 2:** Results of separate linear mixed effects regression models examining effects of activity type on each of language experiences.

		Fixed effects			Random effects		Chi-square test of model fit with and without effect of interest		
Model outcome	Model Parameter	*B*	*SE*	*t*	*Variance*	*SD*	*X*^2^	df	*p*
Peer vocalizations	Activity type	.15	.02	9.27	—	—	85.33	1	< .00001
	Sex	.004	.05	.08	—	—	.008	1	.928
	Age	.0006	.00007	7.71	—	—	57.53	1	< .00001
	Subject intercept	—	—	—	.005	.07	30.54	1	< .00001
Child vocalizations	Activity type	.06	.01	4.33	—	—	18.71	1	< .00001
	Sex	-.07	.05	-1.29	—	—	1.83	1	.177
	Age	.0001	.00007	1.92	—	—	3.77	1	.052
	Subject intercept				.007	.09	128.28	1	< .00001
Adult vocalizations	Activity type	.45	.03	17.61	—	—	302.05	1	< .00001
	Sex	-.10	.06	-1.58	—	—	2.71	1	.10
	Age	.0005	.0001	4.47	—	—	19.04	1	< .00001
	Subject intercept	—	—	—	.009	.10	27.55	1	< .00001
Turn taking	Activity type	.21	.01	21.48	—	—	444.83	1	< .00001
	Sex	-.09	.04	-2.18	—	—	4.70	1	.030
	Age	.0001	.00005	2.65	—	—	7.02	1	.008
	Subject intercept	—	—	—	.004	.0627	157.66	1	< .00001
Overlap	Activity type	.06	.02	2.27	—	—	5.15	1	.023
	Sex	-.02	.02	-.68	—	—	.46	1	.497
	Age	-.00003	.0008	-.33	—	—	.11	1	.74
	Subject intercept	—	—	—	1.6e-15	4e-8	0	1	1

**Note.** 5 models are shown. Chi-square tests contrast models with and without the fixed effect of interest (with all other fixed effects present). Results indicate the robust effect of activity type (structured versus unstructured) on peer vocalizations, child vocalizations, adult vocalization, turn-taking, and vocalization overlap. Family wise alpha = .01.

**Table 3:** Results of linear mixed effects regression models predicting children’s in-the-moment vocalizations based on LENA coded 5-minute bins.

		Fixed effects			Random effects		Chi-square test of model fit with and without effect		
	Model Parameter	*B*	*SE*	*T*	*Variance*	*SD*	*X*^2^	df	*p*
Not including overlap as a covariate	Adult vocalizations	-.20	.006	-34.50	—	—	1116.9	1	< .00001
	Peer vocalizations	.58	.008	72.09	—	—	4074.4	1	< .00001
	Turn-taking	.85	.01	64.53	—	—	3405.5	1	< .00001
	Sex	-.01	.03	-.46	—	—	.28	1	.598
	Age	-.0001	.0001	-.81	—	—	.85	1	.356
	Subject intercept	—	—	—	.002	.04	76.76	1	< .00001
Including overlap as a covariate	Adult vocalizations	-.25	.004	-56.53	—	—	2724.4	1	< .00001
	Peer vocalizations	.17	.008	22.03	—	—	472.62	1	< .00001
	Turn-taking	.82	.01	83.84	—	—	5158.9	1	< .00001
	Overlap	.37	.004	85.59	—	—	5318.9	1	< .00001
	Sex	-.02	.03	-1.37	—	—	.51	1	.476
	Age	-.0002	.0001	-.63	—	—	2.22	1	.136
	Subject intercept	—	—	—	.002	.04	224.38	1	< .00001

**Note.** Adult vocalizations, peer vocalizations, and turn-taking were all robustly associated with children’s vocalizations. Results were not impacted by inclusion of overlap as a predictor. Family wise alpha = .05.

**Table 4:** Results of linear mixed effects regression models predicting children’s daily vocalizations rates.

		Fixed effects			Random effects		Chi-square test of model fit with and without effect		
	Model Parameter	*B*	*SE*	*t*	*Variance*	*SD*	*X*^2^	df	*p*
Including turn-taking as a covariate	Adult vocalizations	-.19	.03	-6.18	—	—	35.31	1	< .00001
	Peer vocalizations	.33	.04	8.12	—	—	56.95	1	< .00001
	Turn-taking	1.24	.08	15.84	—	—	153.20	1	< .00001
	Sex	.10	.23	.42	—	—	.23	1	.628
	Age	-.00009	.0006	-.14	—	—	.03	1	.863
	Subject intercept	—	—	—	.08	.28	1.37	1	.24
Not including turn-taking as a covariate	Adult vocalizations	.10	.04	2.64	—	—	6.99	1	.008
	Peer vocalizations	.25	.06	4.00	—	—	15.13	1	.0001
	Sex	-.54	.52	-1.11	—	—	1.41	1	.235
	Age	-.0001	.001	.09	—	—	.02	1	.882
	Subject intercept	—	—	—	.59	.77	24.83	1	< .00001

**Note.** Adult vocalizations, peer vocalizations, and turn-taking all predict children’s daily vocalization rates. When turn-taking with adults is removed as a predictor, the negative association between adult vocalizing and child vocalizing becomes a positive association. Family wise alpha = .05.

**Table 5:** Results of separate linear mixed effects regression models predicting changes in children’s daily vocalizations over time. Family wise alpha = .0125.

		Fixed effects			Random effects		Chi-square test of model fit with and without effect		
Model Outcome	Model Parameter	*B*	*SE*	*t*			*X*^2^	Df	*p*
Adult vocalizations	Time	.002	.002	1.23	—	—	1.55	1	.213
	Child vocalizations	-1.04	.16	-6.59	—	—	39.85	1	< .00001
	Peer vocalizations	.83	.09	9.32	—	—	71.87	1	< .00001
	Turn-taking	2.50	.21	11.99	—	—	106.16	1	< .00001
	Sex	.26	.37	.71	—	—	.53	1	.468
	Subject intercept	—	—	—	0	0	0	1	1
Turn-taking	Time	.0004	.0005	.93	—	—	.76	1	.384
	Child vocalizations	.50	.03	17.26	—	—	160.97	1	< .00001
	Adult vocalizations	.19	.02	11.91	—	—	103.75	1	< .00001
	Peer vocalizations	-.20	.03	-7.52	—	—	50.29	1	< .00001
	Sex	-.26	.10	-2.43	—	—	6.12	1	.013
	Subject intercept	—	—	—	.003	.05	0	1	1
Child vocalizations	Time	-.001	.008	-1.30	—	—	1.66	1	.20
	Adult vocalizations	-.19	.03	-6.04	—	—	34.05	1	< .00001
	Peer vocalizations	.34	.04	8.45	—	—	61.38	1	< .00001
	Turn-taking	1.24	.08	16.01	—	—	154.72	1	< .00001
	Sex	.10	.23	.45	—	—	.23	1	.632
	Subject intercept	—	—	—	.71	.84	2.81	1	.094
Peer vocalizations	Time	.004	.001	3.61	—	—	11.13	1	.0008
	Child vocalizations	.91	.11	8.49	—	—	62.11	1	< .00001
	Adult vocalizations	.41	.05	9.08	—	—	68.78	1	< .00001
	Turn-taking	-1.31	.18	-7.40	—	—	49.21	1	< .00001
	Sex	.03	.33	.08	—	—	.003	1	.955
	Subject intercept	—	—	—	.11	.33	.04	1	.852

**Table 6:** Results of linear regression models predicting children’s vocabulary growth. Family wise alpha = .05.

	Model Parameter	*B*	*SE*	*T*	*df*	*p*
Using average rates as language input predictors	Child vocalizations	-1.92	.65	-2.96	12	.025
	Adult vocalizations	-1.15	.33	-3.51	12	.013
	Peer vocalizations	1.10	.32	3.41	12	.014
	Turn-taking	3.87	1.21	3.21	12	.018
	Sex	.44	.31	1.42	12	.205
	Age	-.003	.001	-2.21	12	.069
Using change over time as language input predictors	Child vocalizations	-.003	.001	-2.58	12	.042
	Adult vocalizations	-.002	.08	-2.87	12	.028
	Peer vocalizations	.002	.07	3.34	12	.016
	Turn-taking	.007	.003	2.76	12	.033
	Sex	.53	.33	1.61	12	.159
	Age	-.003	.001	-1.89	12	.108

We examined concurrent associations of language input and language use at multiple timescales. Examining associations at multiple timescales involved performing multiple statistical tests whose purpose was to tests probe the robustness of the statistical associations being examined. In addition, we applied a family-wise Bonferroni correction when multiple dependent variables were predicted from the same model at any given time scale. Specific alpha levels, equal to .05 divided by the number of tests in a given family, are noted in each table’s notes.

## Results

The primary study question concerned associations between children’s classroom language input and language use over a 5 minute timescale and a daily timescale, and how these experiences were associated with children’s language development. Specifically, we first asked how changes in classroom activity context over the school day were associated with children’s language input and use (examined in 5-minute increments). Then, we analyzed how children’s language use related to adult vocalizations, peer vocalizations, turn-taking, and overlapping vocalizations within those same 5-minute increments. Next, we analyzed language input and language use over the entire day to determine whether associations between these variables extended beyond the previously analyzed 5-minute increments, which might index language levels within specific conversations. Then, we asked how these measures of daylong experience—children’s language input from their teachers and peers, the number of conversational turns they engaged in with their teachers, and the language they themselves produced—changed over the course of the year. Finally, we examined how yearlong input and use was associated with children’s language development, as index by changes in vocabulary.

### The role of activity context on language input and use

To examine how the different classroom activities related to language, we utilized separate linear mixed effects regression models to predict each of the LENA measures (adult vocalizations, peer vocalizations, child vocalizations, turn-taking, and overlap) from activity type (structured versus unstructured). All models included child sex and age as covariates. See parts M1-5 of the Appendix for full models and Table 2 for full results of each regression analysis.

**Fig 1:**
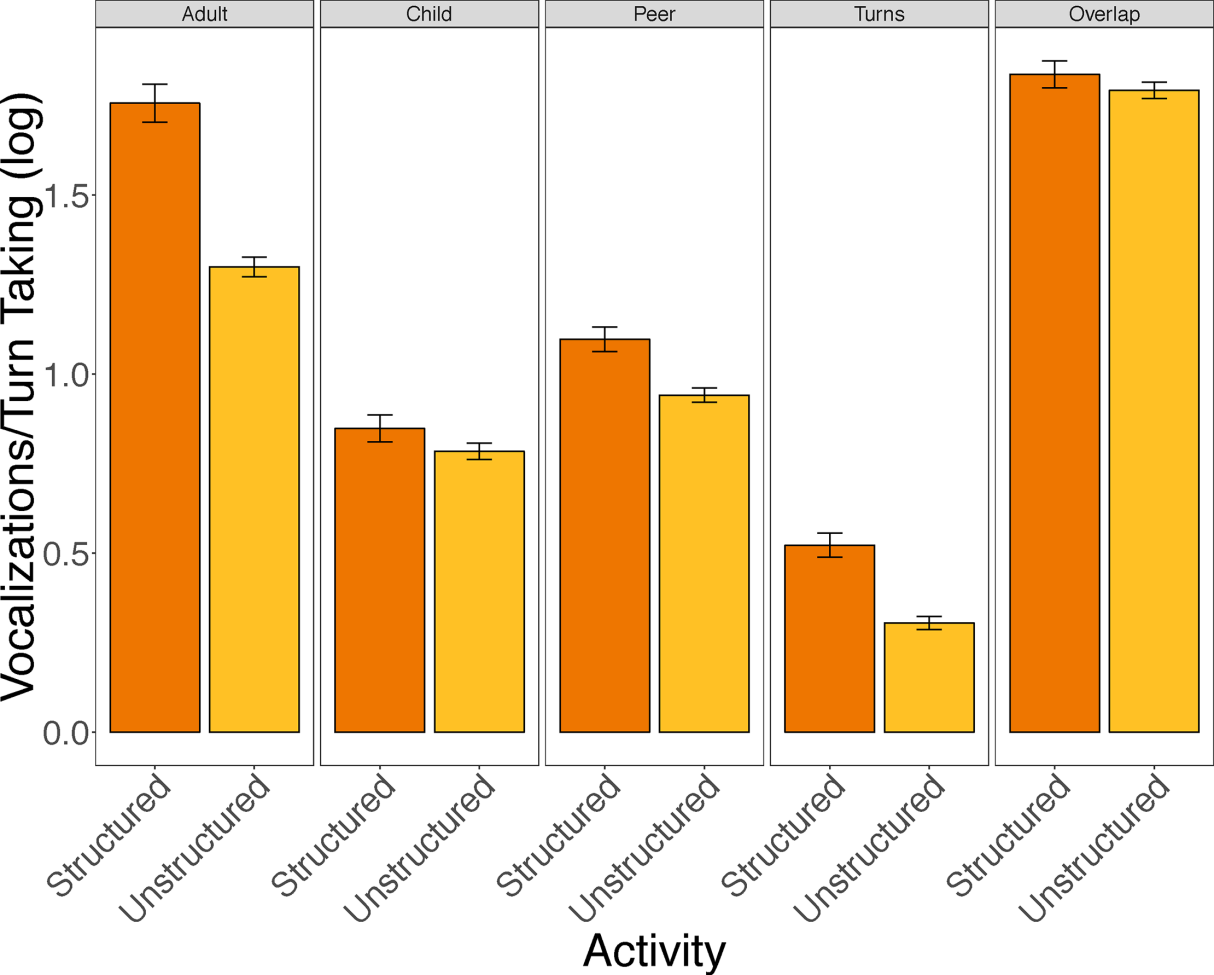
Average number of vocalizations made by adults, children, and peers, average number of conversational turns, and average amount of overlap in vocalizations per 5-minute segment of each type of activity context. Error bars represent standard errors of the mean. See the text for mixed effects models of these effects.

As can be seen in Fig 1, vocalizations and turn-taking varied with activity. Each of the separate models showed that more structured activities were associated with relative increases in each type of vocalization (peer, child, and adult) and turn taking. The model predicting overlap from activity type revealed more overlap during structured than unstructured activities as well. See Table 2 for complete regression tables. Together, these analyses of in-the-moment language input and production indicate that classroom vocalizations are likely to occur during periods of relatively structured activity, such as circle time. We next assess the ways in which the levels of input children receive—regardless of the immediate activity context—relate to their own levels of vocalization.

### Contributions to children’s language use over 5-minute intervals

We predicted children’s vocalizations from peer vocalization, adult vocalization, and turn-taking, including child age and sex as covariates. See Table 3 for results of our regression analysis and Appendix M6 for model structures. To address potential concerns about the collinearity between adult vocalizations and turn-taking, we calculated a variance inflation factor (VIF) for each variable. All VIFs were acceptable with values less than 2.5 [39].

There was a significant relationship between children’s input from peers and their own vocalizations, such that children who vocalized more also tended to receive more input from their peers (see Table 3). Likewise, there was a significant relationship between children’s conversational turn-taking with adults and their own vocalizations, such that children who vocalized more tended to be engaged in more turn-taking. However, children’s input from adults was negatively associated with their own vocalizations, a somewhat surprising result that might reflect temporal competition within 5-minute segments of child and adult vocalizing.

All analyses were replicated while controlling for the LENA measure of talking overlap, which may index imprecision due to classroom noise. Overlap was significantly associated with target child talking. However, its inclusion in models did not impact the robust associations between the language input variables and child talking (see Table 3).

### Contributions to children’s daily language use

We next predicted children’s average rate of vocalizations per hour from their language input from adults and peers and from their turn-taking with adults. This analysis examines the relationships between language input and language use over a longer time-scale not subject to potential conversational dynamics. We included child’s age and sex as covariates. See part M8a of the Appendix for full model and Table 4 for full results of regression analysis.

**Fig 2:**
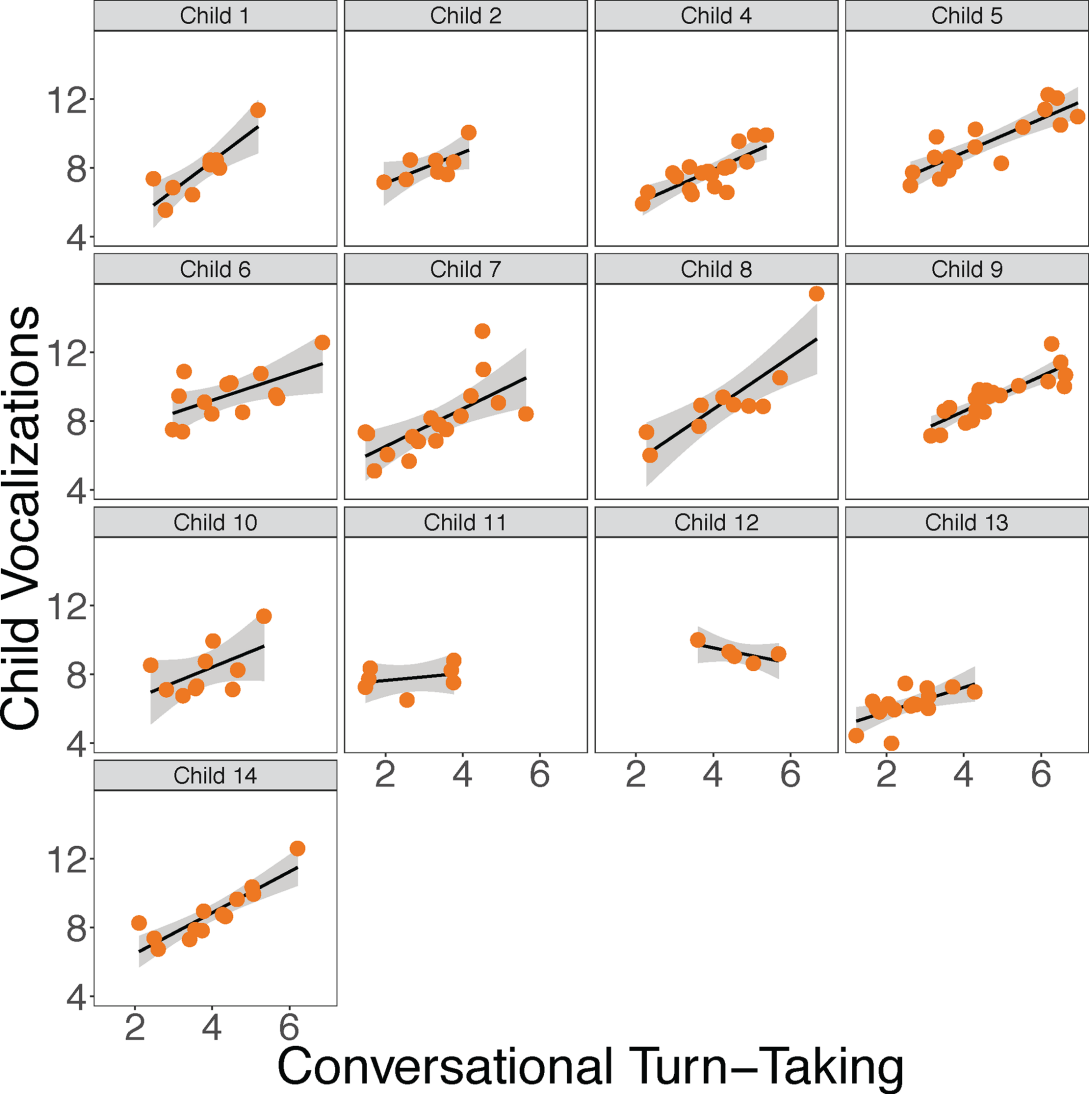
The relationship between each child’s vocalizations and conversational turn-taking. All values are log per hour averages. Each dot represents 1 recording day for 1 child. Error bands represent standard error of the mean.

**Fig 3:**
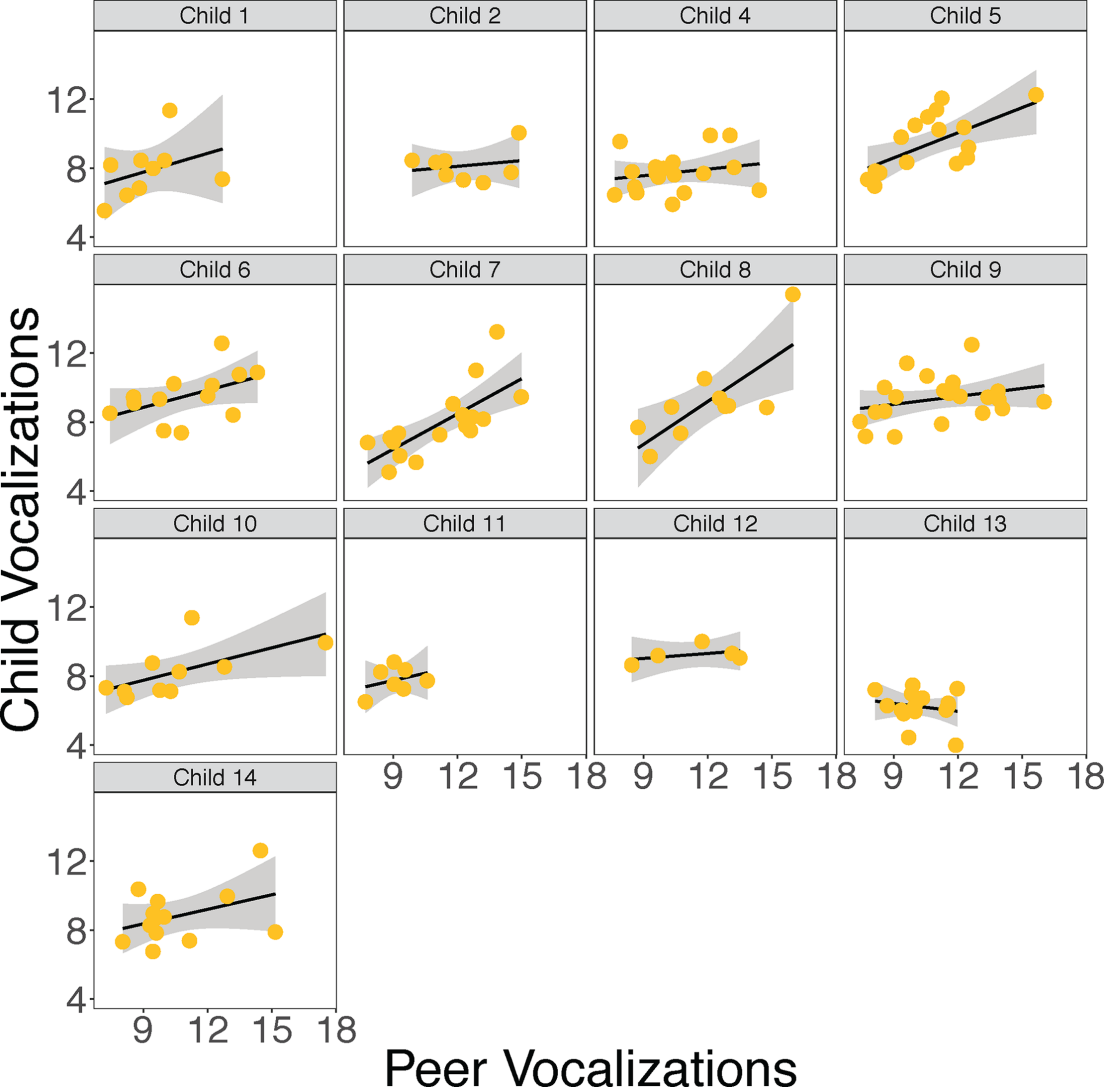
The relationship between each child’s vocalizations and peer vocalizations. All values are log per hour averages. Each dot represents 1 recording day for 1 child. Error bands represent standard error of the mean.

As can be seen in Fig 2, there was a significant association between a child’s average number of conversational turns and their rate of vocalizing, such that children who had more conversational turns tended to vocalize more. See Table 4, See part M8b of the Appendix. As can be seen in Fig 3, we also found a significant positive relationship between the average number of vocalizations children heard from their peers and their own vocalization rate. See Table 4, part M8c of the Appendix. Figs 2 and 3 show the data for each individual child separately. See S1 Fig for all children’s data plotted together.

As in the analyses of 5-minute segments, the daily language model revealed a *negative* relationship between adult input and children’s vocalization levels. See Table 4 and part M8d of the Appendix. However, when conversational turns were not included as a covariate, we found that adult vocalizations were positively associated child vocalizations (see Table 4, parts M9a-d of the Appendix). There is a necessary relationship between the amount of adult vocalizations children hear and the number of conversational turns they engage in with adults—if adults are not vocalizing then there cannot be conversational turns. When we include both variables in our model, we account for separate aspects of their variance in children’s speech. The findings therefore suggest that the component of adult input that is most relevant for enhancing children’s vocalizations is exchanging conversational turns with the adult.

### Changes in language experiences over time

**Fig 4:**
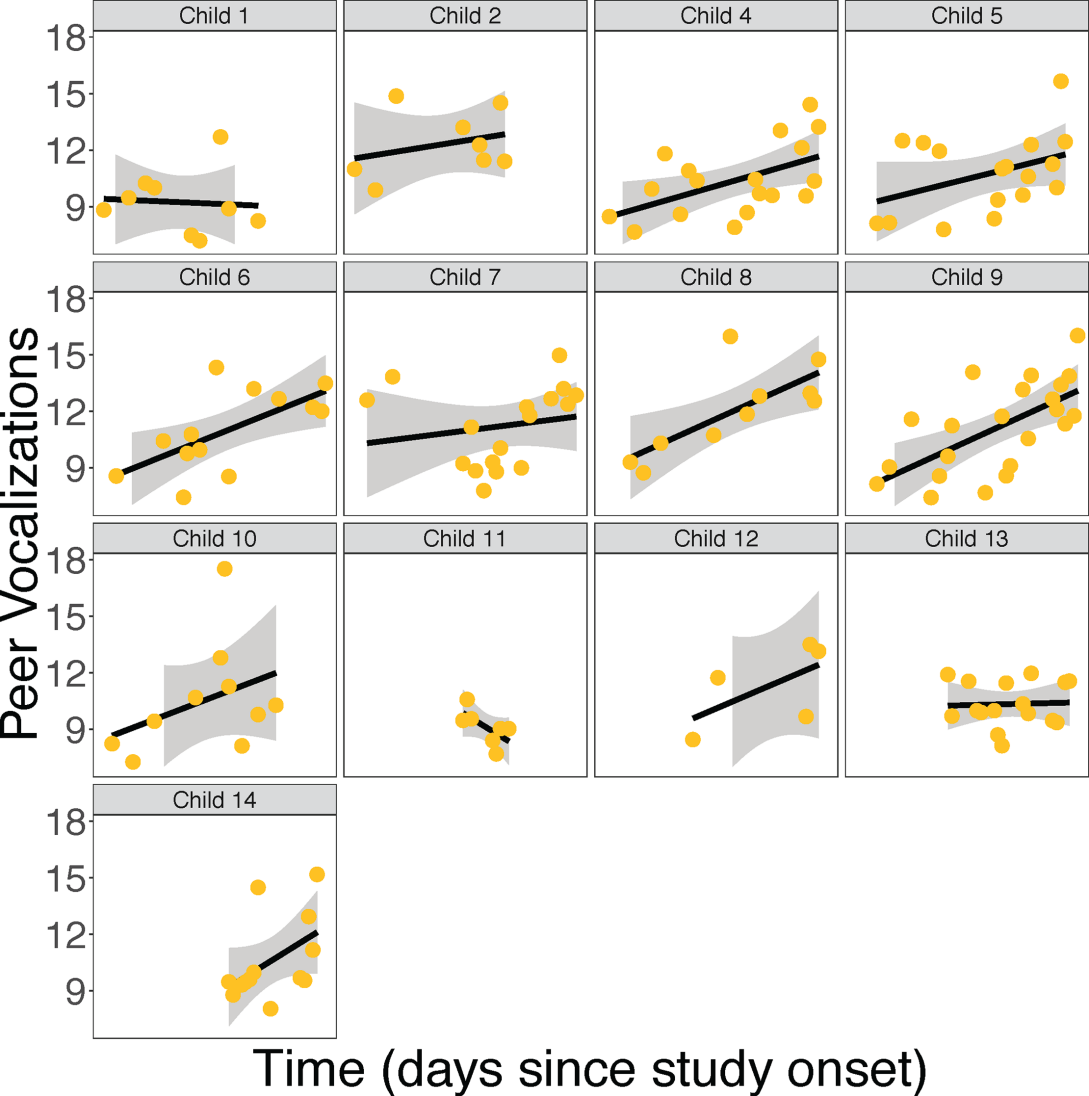
The relationship between time and the number of vocalizations children heard from their peers (log per hour averages). Error bands represent standard error of the mean. Each point represents 1 recording day for 1 child.

To determine the extent to which language experiences increased over time, we created 4 linear mixed regression models that predicted daily levels of child vocalizations, peer vocalizations, adult vocalizations, and turn-taking from their recording date. Each of these models also included sex and each of the other LENA measures of interest as covariates. See parts M10a-13a of the Appendix for full model descriptions and Table 5 for full results of regression analyses. As can be seen in Table 5, neither adult talking (See part M10 of the Appendix), conversational turn-taking (See part M11 of the Appendix), or child talking (See part M12 of the Appendix) increased over the course of the year. However, as can be seen in Table 5 and Fig 4, there was a significant increase in the amount of language children heard from their peers (See part M13 of the Appendix). Fig 4 shows the data for each individual child separately. See S2 Fig for all children’s data plotted together.

Together, these results indicate that although children’s overall amount of vocalization did not increase over the course of the year, the amount of vocalizing they directed to their peers did increase. We return to this issue in the discussion.

### Contributions to children’s language development

Finally, we examined the role of language experiences in children’s language development. To compare developmental changes in children’s language, we assessed the rate of growth in children’s expressive vocabulary size as measured by the MCDI at the beginning, middle, and end of the study. We calculated an average rate of vocabulary growth for each participant by dividing the difference in vocabulary size between assessments by the number of days between assessments. All children contributed 2 or 3 assessments (see Table 1). If a participant contributed to all 3 MCDI assessments, their score was calculated by averaging the 2 rates. If a participant contributed only 2 MCDI assessments, their score was the rate of change between those scores.

We used linear regression to predict the rate of vocabulary change from LENA measures of the daily input from adults, input from other children, number of conversational turns, and children’s own vocalizations (See part M14a of the Appendix). We included sex and age as covariates. As seen in Table 6, there was significant effect of conversational turns on vocabulary growth such that children engaging in the most conversational turns gained vocabulary knowledge at a faster rate than children engaging in fewer conversational turns (See part M14b of the Appendix). Simultaneously the amount of language children heard from their peers significantly predicted their vocabulary growth, such that children hearing more input from other children gained vocabulary at a faster rate than children hearing less input from other children (see Table 6, part M14c of the Appendix).

As in the analyses of child vocalizations at each timescale, we found a negative effect of adult talking on vocabulary growth such that children hearing more input from adults gained vocabulary at a slower rate than children hearing less input from adults (see Table 6, part M14d of the Appendix). Relatedly, children’s own vocalizations were negatively predictive of their rate of vocabulary growth, such that those children who vocalized the most had the slowest rates of vocabulary growth (see Table 6, part M14e of the Appendix). It is critical to note here, however, that our models include conversational turns as a covariate. Thus, models accounted for the separate roles of overall vocalizations and vocalizations within the context of a back and forth conversation in predicting child vocalizations. When we removed conversational turns as a predictor from this model, neither the number of vocalizations children heard from adults, nor the number of vocalizations children produced were associated with vocabulary growth.

Next, we repeated these analyses using measures of vocal change over the course of the year as predictors. Specifically, we used each subject’s slope coefficients from the models predicting LENA measures over time (See part M15a of the Appendix). This model assessed the extent to which increases in language input/use over time was associated with vocabulary growth. There were no differences between models with respect to the significance of predictors (See part M14 of the Appendix and Table 6). Of specific interest, increases in peer vocalizations and turn–taking over the course of the year were associated with increases in expressive vocabulary.

## Discussion

The study goal was to use day-long recordings of individual children in an early intervention preschool classroom to understand the language experiences that predict children’s language use and development over multiple timescales. The input children received from their peers was associated with their own language use both over 5-minute intervals and over the course of a day; and peer input was associated with increases in vocabulary over the course of a year. Likewise, teacher-child turn-taking was associated with increases in children’s vocalizations over 5-minute intervals and over the course of a day and with increases in vocabulary over the course of the year. Adult vocalizations that did not occur in the context of turn-taking were negatively associated with children’s language at every timescale. We discuss the implications of each of these findings and directions for future investigation below.

### The role of peers’ language use on language development

In this study, peer input was strongly associated with children’s language use and development across time scales. Children who received more input from peers vocalized more both over 5-minute intervals and over the course of the day. Moreover, children who heard more vocalizations from peers had higher rates of vocabulary acquisition than children who heard fewer vocalizations from peers.

Interacting with a more advanced peer can provide a child with opportunities to hear new or unknown vocabulary and grammatical constructions, similar to interacting with an adult. Indeed, children with initially poorer language abilities benefit more from their peers (as measured by the average language ability of the class as a whole) over the course of a school year than children with better initial language abilities (see e.g., [24,40]). Within a given classroom, however, not every child can interact with a more advanced peer. Nevertheless, contagion theories of peer influences suggest that a high ability peer can sometimes influence other children’s abilities, without being influenced themselves by those lower ability peers (e.g., [41]). Higher ability peers may specifically benefit from explaining and being forced to consider and frame explanations within the lower ability peer’s zone of proximal development (cf [41,42]). In the current study, we were unable to assess which specific peers the target child interacted with and what they learned from those interactions. A critical direction for future research will therefore be to assess how interactions with specific peers influence language and social outcomes. Such a study would have important implications for questions of inclusion for children with language delays or communication disorders.

It is noteworthy that peer vocalizations, but not child vocalizations, increased over the course of the year. This suggests that children did not change their overall rates of vocalizations, but rather changed to whom they directed these vocalizations. It is not clear what could have driven this increase, as a linear regression model revealed that there was not a significant increase in students present in the classroom over the year (p = .12). Instead, the increase in peer vocalizations suggests that there may have been concurrent changes to children’s social interaction abilities. Future work will explore this link between changes in children’s social skills and their partnered vocalizations.

### The role of turn-taking on language development

Our results extend previous findings illustrating the impact of turn-taking in a classroom context. Earlier work examined teachers’ language quality as a stable property of the adult [43–45]. The current results indicate that input quality varies dynamically as a function of activity context and child. Critically, that variability is related to how much children vocalize in the moment, in a day, and for vocabulary development over the course of a year. The association between turn-taking and children’s vocalizations within 5-minute increments is likely indicative of a dynamic elicitation of child vocalizations within a conversation. The more long-range association between turn-taking and child vocalizations over the course of a day suggests this elicitation may ‘scale up’ to influence children’s overall levels of language production.

The association between turn-taking and children’s vocabulary growth is consistent with the idea that there are pervasive effects of conversation on development. The back and forth nature of conversation also allows not just for more talking opportunities, but also for richer *learning* opportunities. When an adult engages a child in conversation, that engagement draws them in, increasing attention to the adult’s words and their meanings, and provides the child opportunities to use language constructively (cf [46]). For example, bilingual toddlers’ own language use influences their subsequent vocabulary growth [47]. The attention required to understand and respond topically to an adult’s utterance may scaffold the acquisition of new words, leading to faster rates of vocabulary development. It is not enough to simply talk around children; instead adults need to talk *with* children. Adult-child *conversation*, rather than adult vocalizations directed at a child, is often characterized by the adult’s use of expansions on what the child has just said and speech that elicits further responding. Previous work directly assessing these types of constructions demonstrates that adults’ use of open-ended questions and other methods that encourage the child to respond have a positive effect on vocabulary acquisition in preschool-aged children [48–51].

It is possible that conversational turn-taking may index an increase in caregiver’s sensitivity to the child’s focus of attention and responsivity to the developmental level of their vocalization [51,52]. If so, the content of what an adult says in the context of shared attention and conversation could be more relevant and more learnable to the child than the content of adult vocalizations outside of shared attention and conversation [53,54]. Critically, our current approach means that we do not know the specific content of adult-child conversations or the extent to which conversations did index shared attention. Future classroom research will be critical to understanding what happens in bouts of turn-taking and the mechanisms by which it facilitates children’s language development.

### Potential limitations

#### Interpreting directionality

The unobtrusiveness of the LENA recorders allowed us to effectively capture forty-2 representative weeks of life in this classroom. However, the observational design does not indicate the direction of potential causal associations between teachers’, peers’ and children’s own levels of talking. Teachers may have engaged in more turn-taking with children who spoke more, or turn-taking may have led children to talk more. Both possibilities are likely. Critically however, analyses of vocabulary growth controlled for children’s own levels of vocalization. In these analyses, children who engaged in the most turn-taking with teachers and received the most input from peers exhibited the highest levels of vocabulary growth. Although future experimental work will help to tease apart these associations, the current findings are consistent the proposal that peer input and conversational turn-taking with a teacher each facilitated vocabulary development.

#### Sample size

The study intensely examined language use and development over the course of a year within 1 classroom. Having a small number of participants with a large number of data points per participant is consistent with other recent studies of the dynamics of children’s learning experiences, which also employ linear mixed effects models (cf [53]). Much previous research work comparing multiple classrooms involved more individual participants than the current study did but obtained fewer individual data points from each participant. Classrooms were typically treated statically, with a single number used to describe the teacher’s vocal input and a single number used to describe classroom performance. The current perspective does not consider viewed the classroom as a static whole, but instead takes advantage of first-person audio from individual children to understand the dynamics of language experiences within a classroom. In selecting a single classroom of children in the targeted age range to study, class size was an inherent limitation. Although analyses tested the robustness of associations at multiple levels of analysis, additional research is necessary to understand the generalizability of the current results.

### Conclusions

In the current study, we employed a big data approach of lengthy, repeated measurement of individual children’s language milieus. The application of new recording and analysis technology to quantifying language experiences in the classroom provides new insight into the dynamics of the classroom language environment and their consequences for language in at-risk children. Together the current results indicate the importance of classroom activity context and language experiences, especially input from peers and conversational turn-taking with adults, in facilitating language development. These findings suggest specific contextual factors associated with language gains in high-risk children from low-SES families who may receive less than optimal levels of quality language input at home.

## Appendix

### Regression models used in each analysis

#### The role of activity context on language input and use

childLOG = log_10_ (1 + number of target child vocalizations in each 5 minute period of recording)

adultLOG = log_10_ (1 + number of adult vocalizations in each 5 minute period of recording)

peerLOG = log_10_ (1 + number of non-target child vocalizations in each 5 minute period of recording)

turnLOG = log_10_ (1 + number of conversational turns between target child and adult in each minute period of recording)

overlapLOG = log_10_ (1 + number of instances of overlap in each 5 minute period of recording)

Child_Gender = male/female

Child_AgeDays = age of target child at each recording

(1|subjCode) = random intercept for each subject

#### Amount of peer talking

M1a <- lmer (peerLOG ~ activityCode + childLOG + turnLOG + adultLOG + Child_Gender + Child_AgeDays + (1|subjCode), data = d)

M1b <- lmer (peerLOG ~ 1 + Child_Gender + Child_AgeDays + (1|subjCode), data = d)

M1c <- lmer (peerLOG ~ activityCode + 1 + Child_AgeDays + (1|subjCode), data = d)

M1d <- lmer (peerLOG ~ activityCode + Child_Gender + 1 + (1|subjCode), data = d)

#### Amount of child talking

M2a <- lmer (childLOG ~ activityCode + Child_Gender + Child_AgeDays + (1|subjCode), data = d)

M2b <- lmer (childLOG ~ 1 + Child_Gender + Child_AgeDays + (1|subjCode), data = d)

M2c <- lmer (childLOG ~ activityCode + 1 + Child_AgeDays + (1|subjCode), data = d)

M2d <- lmer (childLOG ~ activityCode + Child_Gender + 1 + (1|subjCode), data = d)

#### Amount of adult talking

M3a <- lmer (adultLOG ~ activityCode + Child_Gender + Child_AgeDays + (1|subjCode), data = d)

M3b <- lmer (adultLOG ~ 1 + Child_Gender + Child_AgeDays + (1|subjCode), data = d)

M3c <- lmer (adultLOG ~ activityCode + 1 + Child_AgeDays + (1|subjCode), data = d)

M3d <- lmer (adultLOG ~ activityCode + Child_Gender + 1 + (1|subjCode), data = d)

#### Amount of turn-taking

M4a <- lmer (turnLOG ~ activityCode + Child_Gender + Child_AgeDays + (1|subjCode), data = d)

M4b <- lmer (turnLOG ~ 1 + Child_Gender + Child_AgeDays + (1|subjCode), data = d)

M4c <- lmer (turnLOG ~ activityCode + 1 + Child_AgeDays + (1|subjCode), data = d)

M4d <- lmer (turnLOG ~ activityCode + Child_Gender + 1 + (1|subjCode), data = d)

#### Amount of overlap

M5a <- lmer (overlapLOG ~ activityCode + Child_Gender + Child_AgeDays + (1|subjCode), data = d)

M5b <- lmer (overlapLOG~ 1 + Child_Gender + Child_AgeDays + (1|subjCode), data = d)

M5c <- lmer (overlapLOG~ activityCode + 1 + Child_AgeDays + (1|subjCode), data = d)

M5d <- lmer (overlapLOG~ activityCode + Child_Gender + 1 + (1|subjCode), data = d)

### Contributions to children’s language use over 5-minute intervals

M6a <-lmer (childLOG ~ adultLOG + peerLOG + turnLOG + Child_Gender + Child_AgeDays + (1|subjCode), data = d)

M6b <-lmer (childLOG ~ adultLOG + 1 + turnLOG + Child_Gender + Child_AgeDays + (1|subjCode), data = d)

M5c <-lmer (childLOG ~ 1 + peerLOG + turnLOG + Child_Gender + Child_AgeDays + (1|subjCode), data = d)

M6d <-lmer (childLOG ~ adultLOG + peerLOG + 1 + Child_Gender + Child_AgeDays + (1|subjCode), data = d)

M6e <-lmer (childLOG ~ adultLOG + peerLOG + turnLOG + 1 + Child_AgeDays + (1|subjCode), data = d)

M6f <-lmer (childLOG ~ adultLOG + peerLOG + turnLOG + Child_Gender + 1 + (1|subjCode), data = d)

#### Including overlap as a covariate

M7a <-lmer (childLOG ~ adultLOG + peerLOG + turnLOG + Child_Gender + Child_AgeDays + overlapLOG + (1|subjCode), data = d)

M7b <-lmer (childLOG ~ adultLOG + 1 + turnLOG + Child_Gender + Child_AgeDays + overlapLOG + (1|subjCode), data = d)

M7c <-lmer (childLOG ~ 1 + peerLOG + turnLOG + Child_Gender + Child_AgeDays + overlapLOG + (1|subjCode), data = d)

M7d <-lmer (childLOG ~ adultLOG + peerLOG + 1 + Child_Gender + Child_AgeDays + overlapLOG + (1|subjCode), data = d)

M7e <-lmer (childLOG ~ adultLOG + peerLOG + turnLOG + 1 + Child_AgeDays + overlapLOG + (1|subjCode), data = d)

M7f <-lmer (childLOG ~ adultLOG + peerLOG + turnLOG + Child_Gender + 1 + overlapLOG + (1|subjCode), data = d)

M7g <-lmer (childLOG ~ adultLOG + peerLOG + turnLOG + Child_Gender + Child_AgeDays + 1 + (1|subjCode), data = d)

### Contributions to children’s daily language use

childProp = sum of all target child vocalizations each day divided by length of recording

adultProp = sum of all adult vocalizations each day divided by length of recording

peerProp = sum of all non-target child vocalizations each day divided by length of recording

turnProp = sum of all target child vocalizations each day divided by length of recording

M8a <-lmer (childProp ~ adultProp + peerProp + turnProp + Child_Gender + Child_AgeDays + (1|subjCode), data = df)

M8b <-lmer (childProp ~ adultProp + peerProp + 1 + Child_Gender + Child_AgeDays + (1|subjCode), data = df)

M8c <-lmer (childProp ~ adultProp + 1 + turnProp + Child_Gender + Child_AgeDays + (1|subjCode), data = df)

M8d <-lmer (childProp ~ 1 + peerProp + turnProp + Child_Gender + Child_AgeDays + (1|subjCode), data = df)

M8e <-lmer (childProp ~ adultProp + peerProp + turnProp + 1 + Child_AgeDays + (1|subjCode), data = df)

M8f <-lmer (childProp ~ adultProp + peerProp + turnProp + Child_Gender + 1 + (1|subjCode), data = df)

### Contributions to children’s daily language use (not including turn-taking as covariate)

M9a <-lmer (childProp ~ adultProp + peerProp + Child_Gender + Child_AgeDays + (1|subjCode), data = df)

M6b <-lmer (childProp ~ 1 + peerProp + Child_Gender + Child_AgeDays + (1|subjCode), data = df)

M9c <-lmer (childProp ~ adultProp + 1 + Child_Gender + Child_AgeDays + (1|subjCode), data = df)

M9d <-lmer (childProp ~ adultProp + peerProp + 1 + Child_AgeDays + (1|subjCode), data = df)

M9e <-lmer (childProp ~ adultProp + peerProp + Child_Gender + 1 + (1|subjCode), data = df)

### Changes in language experiences over time

studyTime = number of days between recording date and study initiation

#### Amount of adult talking

M10a <-lmer (adultProp ~ studyTime + childProp + peerProp + turnProp + Child_Gender + (1|subjCode), data = df)

M10b <-lmer (adultProp ~ 1 + childProp + peerProp + turnProp+ Child_Gender + (1|subjCode), data = df)

M10c <-lmer (adultProp ~ studyTime + 1 + peerProp + turnProp + Child_Gender + (1|subjCode), data = df)

M10d <-lmer (adultProp ~ studyTime + childProp + 1 + turnProp + Child_Gender + (1|subjCode), data = df)

M10e <-lmer (adultProp ~ studyTime + childProp + peerProp + 1 + Child_Gender + (1|subjCode), data = df)

M10f <-lmer (adultProp ~ studyTime + childProp + peerProp + turnProp + 1 + (1|subjCode), data = df)

#### Amount of turn-taking

M11a <-lmer (turnProp ~ studyTime + adultProp + peerProp + childProp + Child_Gender + (1|subjCode), data = df)

M11b <-lmer (turnProp ~ 1 + adultProp + peerProp + childProp + Child_Gender + (1|subjCode), data = df)

M11c <-lmer (turnProp ~ studyTime + 1 + peerProp + childProp + Child_Gender + (1|subjCode), data = df)

M11d <-lmer (turnProp ~ studyTime + adultProp + 1 + childProp + Child_Gender + (1|subjCode), data = df)

M11e <-lmer (turnProp ~ studyTime + adultProp + peerProp + 1 + Child_Gender + (1|subjCode), data = df)

M11f <-lmer (turnProp ~ studyTime + adultProp + peerProp + childProp + 1 + (1|subjCode), data = df)

#### Amount of child talking

M12a <-lmer (childProp ~ studyTime + adultProp + peerProp + turnProp + Child_Gender + (1|subjCode), data = df)

M12b <-lmer (childProp ~ 1 + adultProp + peerProp + turnProp+ Child_Gender + (1|subjCode), data = df)

M12c <-lmer (childProp ~ studyTime + 1 + peerProp + turnProp + Child_Gender + (1|subjCode), data = df)

M12d <-lmer (childProp ~ studyTime + adultProp + 1 + turnProp + Child_Gender + (1|subjCode), data = df)

M12e <-lmer (childProp ~ studyTime + adultProp + peerProp + 1 + Child_Gender + (1|subjCode), data = df)

M12f <-lmer (childProp ~ studyTime + adultProp + peerProp + turnProp + 1 + (1|subjCode), data = df)

#### Amount of peer talking

M13a <-lmer (peerProp ~ studyTime + adultProp + childProp + turnProp + Child_Gender + (1|subjCode), data = df)

M13b <-lmer (peerProp ~ 1 + adultProp + childProp + turnProp+ Child_Gender + (1|subjCode), data = df)

M13c <-lmer (peerProp ~ studyTime + 1 + childProp + turnProp + Child_Gender + (1|subjCode), data = df)

M13d <-lmer (peerProp ~ studyTime + adultProp + 1 + turnProp + Child_Gender + (1|subjCode), data = df)

M13e <-lmer (peerProp ~ studyTime + adultProp + childProp + 1+ Child_Gender + (1|subjCode), data = df)

M13f <-lmer (peerProp ~ studyTime + adultProp + childProp + turnProp + 1 + (1|subjCode), data = df)

### Contributions to children’s language development

averageRate = average rate of vocabulary growth between time 1 and 2 and time 2 and 3

turns = average daily rate of conversational turns for each child for the entire year

child = average daily rate of each child’s vocalizations for the entire year

adult = average daily rate of adult input for each child for the entire year

peer = average daily rate of peer input for each child for the entire year

Child_DOB = each child’s date of birth, to capture differences in age

turnsChange = change in daily rate of conversational turns for each child over the entire year

childChange = change in daily rate of each child’s vocalizations over the entire year

adultChange = change in daily rate of adult input for each child over the entire year

peerChange = change in daily rate of peer input for each child over the entire year

#### Using averages of LENA measures as language experience predictors

M14a <-lm(averageRate ~ turns + child + adult + peer + Child_Gender + Child_DOB, data = l)

M14b <-lm(averageRate ~ 1 + child + adult + peer + Child_Gender + Child_DOB, data = l)

M14c <-lm(averageRate ~ turns + child + adult + 1 + Child_Gender + Child_DOB, data = l)

M14d <-lm(averageRate ~ turns + child + 1 + peer + Child_Gender + Child_DOB, data = l)

M14e <-lm(averageRate ~ turns + 1 + adult + peer + Child_Gender + Child_DOB, data = l)

M14f <-lm(averageRate ~ turns + child + adult + peer + 1 + Child_DOB, data = l)

M14g <-lm(averageRate ~ turns + child + adult + peer + Child_Gender + 1, data = l)

#### Using changes to LENA measures over time as language experience predictors

M15a <-lm(averageRate ~ turnsChange + childChange + adultChange + peerChange + Child_Gender + Child_DOB, data = l)

M15b <-lm(averageRate ~ 1 + childChange + adultChange + peerChange + Child_Gender + Child_DOB, data = l)

M15c <-lm(averageRate ~ turnsChange + childChange + adultChange + 1 + Child_Gender + Child_DOB, data = l)

M15d <-lm(averageRate ~ turnsChange + childChange + 1 + peerChange + Child_Gender + Child_DOB, data = l)

M15e <-lm(averageRate ~ turnsChange + 1 + adultChange + peerChange + Child_Gender + Child_DOB, data = l)

M15f <-lm(averageRate ~ turnsChange + childChange + adultChange + peerChange + 1 + Child_DOB, data = l)

M15g <-lm(averageRate ~ turnsChange + childChange + adultChange + peerChange + Child_Gender + 1, data = l)

## Supporting information

S1 Fig The relationship between children’s vocalizations and conversational turns (A) and peer vocalizations (B). All vocalizations and turn-taking values are log per hour averages. Error bands represent standard error of the mean. Each point represents 1 recording day for 1 child.
(PDF)

S2 Fig The relationship between time and the number of vocalizations children heard from their peers (log per hour averages). Error bands represent standard error of the mean. Each point represents 1 recording day for 1 child.
(PDF)
