# Supplementary figures and images for: A year in words: The dynamics and consequences of language experiences in an intervention classroom
Source: PLoS One. 2018 Jul 6;13(7):e0199893. doi: 10.1371/journal.pone.0199893 (PMC6034821; doi:10.1371/journal.pone.0199893)

A

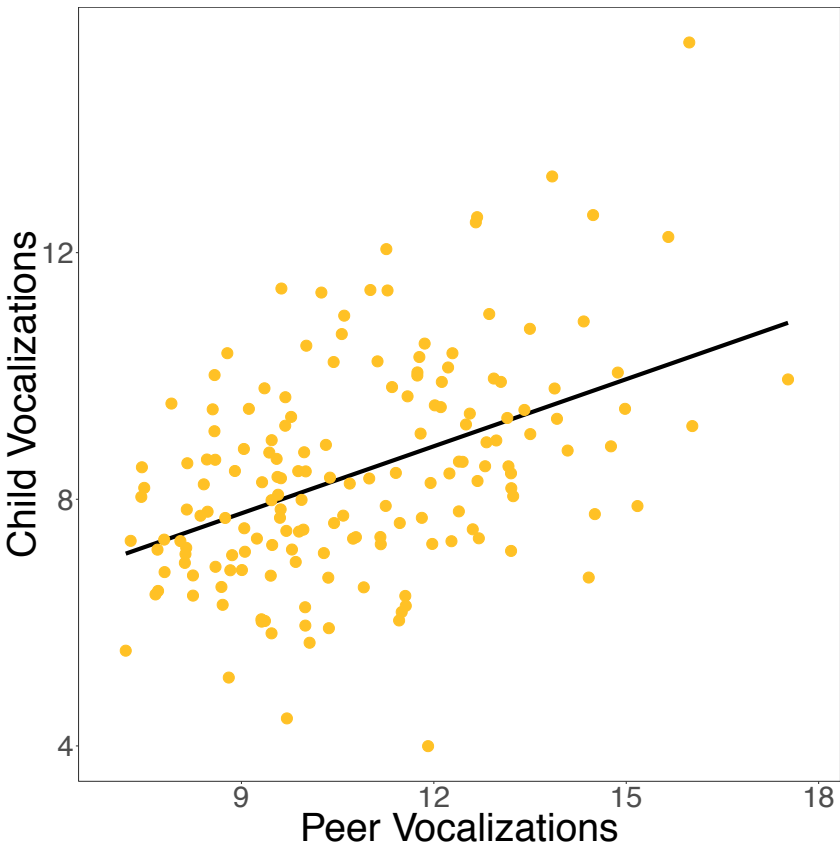

B

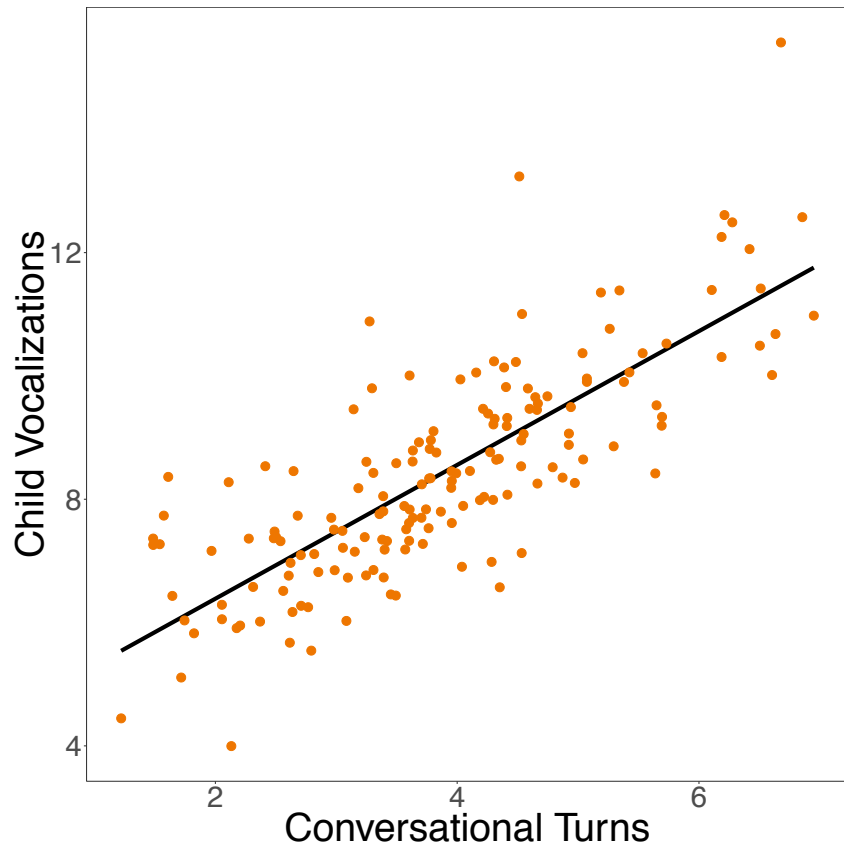

Supplement: S1 Fig — The relationship between children’s vocalizations and conversational turns (A) and peer vocalizations (B). All vocalizations and turn-taking values are log per hour averages. Error bands represent standard error of the mean. Each point represents 1 recording day for 1 child. (PDF) [file pone.0199893.s001.pdf]

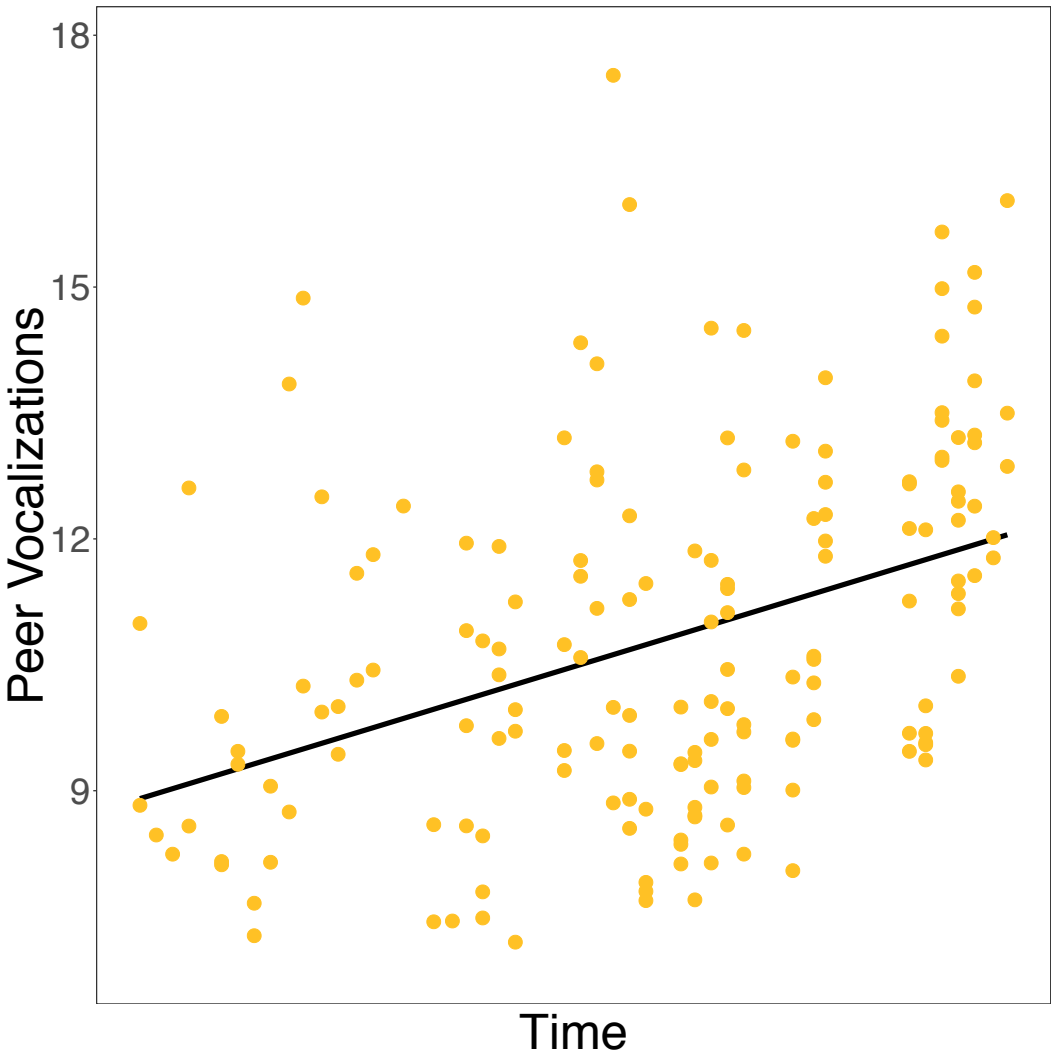

Supplement: S2 Fig — Error bands represent standard error of the mean. Each point represents 1 recording day for 1 child. (PDF) [file pone.0199893.s002.pdf]
